# Efficacy of Optical Internal Urethrotomy and Intralesional Injection of Vatsala-Santosh PGI Tri-Inject (Triamcinolone, Mitomycin C, and Hyaluronidase) in the Treatment of Anterior Urethral Stricture

**DOI:** 10.1155/2014/192710

**Published:** 2014-10-01

**Authors:** Santosh Kumar, Nitin Garg, Shrawan Kumar Singh, Arup Kumar Mandal

**Affiliations:** Department of Urology, PGIMER, Chandigarh 160012, India

## Abstract

*Purpose*. To study the efficacy of optical internal urethrotomy with intralesional injection of Vatsala-Santosh PGI tri-inject (triamcinolone, mitomycin C, and hyaluronidase) in the treatment of anterior urethral stricture. *Material and Methods*. A total of 103 patients with symptomatic anterior urethral stricture were evaluated on the basis of clinical history, physical examination, uroflowmetry, and retrograde urethrogram preoperatively. All patients were treated with optical internal urethrotomy followed by injection of tri-inject at the urethrotomy site. Tri-inject was prepared by diluting the combination of triamcinolone 40 mg, mitomycin C 2 mg, and hyaluronidase 3000 in 5–10 mL of saline according to length of stricture. An indwelling 18 Fr silicone catheter was left in place for a period of 7–21 days. All patients were followed up for 6–18 months postoperatively on the basis of history, uroflowmetry, and, if required, retrograde urethrogram and micturating urethrogram every 3 months. *Results*. The overall recurrence rate after first OIU is 19.4% (20 out of 103 patients), that is, a success rate of 80.6%. Overall recurrence rate after second procedure was 5.8% (6 out of 103 patients), that is, a success rate of 94.2%. *Conclusion*. Optical internal urethrotomy with intralesional injection of Vatsala-Santosh PGI tri-inject (triamcinolone, mitomycin C, and hyaluronidase) is a safe and effective minimally invasive therapeutic modality for short segment anterior urethral strictures.

## 1. Introduction

Urethral stricture disease has always been a challenge for urologists. Different treatment modalities that are used for treatment of urethral stricture disease are dilatation, urethrotomy, stent placement, and urethroplasty. Steenkamp et al. have found no significant difference in efficacy between dilation and internal urethrotomy as initial treatment of strictures [1]. Internal urethrotomy is a safe first line treatment for urethral strictures independent of etiology and location, with an overall primary success rate of 60–70% [2]. Endoscopic treatment is recommended before various forms of urethroplasty are contemplated [2]. Pansadoro and Emiliozzi [3] have shown that the curative success rate of direct visual internal urethrotomy (DVIU) is approximately 30 to 35%. The low success rate and the recurrence of stricture despite treatment have prompted the search for new treatment methods. Ho:YAG laser urethrotomy is a safe and effective minimally invasive therapeutic modality for urethral stricture with results comparable to those of conventional urethrotomy [4]. In intervention for recurrent urethral stricture holmium laser treatment is safe and effective [5]. Application of steroid at time of urethrotomy produces better result than urethrotomy alone [5, 6]. Mitomycin C is useful in delaying the healing process by preventing replication of fibroblasts and epithelial cells and inhibiting collagen synthesis. It is also proposed that it can delay wound contraction [7]. Hyaluronidase instillation during OIU may decrease the incidence of urethral stricture recurrence [8]. The exact mechanism is not known in urethral stricture but it is used as antifibrotic agent in hypertrophic scar, keloid, and pulmonary fibrosis. Intralesional injection decreases fibroblast proliferation, collagen, and glycosaminoglycan synthesis and suppresses proinflammatory mediators in wound healing process [9]. Thus this study was conducted to see the benefit of combining all these three agents for preventing recurrence of stricture after urethrotomy. The term Vatsala-Santosh PGI tri-inject refers to the name of the investigators and institution where the work was carried out.

## 2. Material and Methods

A total of 103 patients with symptomatic anterior urethral stricture (primary or secondary) were treated by optical internal urethrotomy followed by intralesional injection of Vatsala-Santosh PGI tri-inject during a period from December 2011 to June 2013 in PGIMER, Chandigarh. The study was approved by the institute ethical committee and informed consent was taken from the patients before enrolment in the study. Patients with completely obliterated urethral stricture were excluded from the study. Patients presenting for the first time for treatment were referred to as primary, whereas those who had undergone some procedure for the treatment of stricture prior to reporting to us were referred to as secondary. Diagnosis of urethral stricture was made on the basis of clinical history, uroflowmetry, and retrograde urethrography. Patients were categorised into three groups depending upon the location of the stricture: penile, bulbar, and pan-anterior. Patients with bulbar urethral stricture were further categorised depending upon the length of stricture (<2 cm, 2–4 cm, and >4 cm) and urethral calibration (<6 Fr and ≥6 Fr). The procedure was done under general or regional anesthesia. All patients received antibiotic prophylaxis preoperatively. Optical internal urethrotomy was done in usual manner using cold knife. Tri-inject was prepared by diluting triamcinolone 40 mg, mitomycin 2 mg, hyaluronidase 3000 U in 5–10 mL of saline according to length of stricture and was injected intralesionally at the site of urethrotomy using William's endoscopic needle. At every site 1-2 mL was injected. After confirming free passage of cystoscope into the bladder, an 18 Fr silicone catheter was left in place for 7–21 days. Culture specific broad spectrum antibiotics were administered perioperatively and continued till catheter removal. Postprocedure evaluation was done on the basis of history and uroflowmetry. Retrograde urethrography and micturating cystourethrography were done only if patient developed obstructive voiding problems or flow rate below 10 mL/second. Follow up was done at regular interval of 1 month. Any symptoms pertaining to recurrence were noted as reduced stream of urine, retention of urine, and burning micturition. Procedure was considered successful if patient did not report any voiding difficulty and maximum flow rate >10 mL/second for a voided volume of at least 100 mL.

## 3. Results

Median followup was 14 months (3–18 months) and median age at presentation was 47 years (17–80 years). Of these 103 patients, 80 (77.66%) had bulbar urethral stricture, 7 (6.8%) had pendular urethral stricture, and 16 (15.5%) patients had pan-anterior urethral stricture.

The baseline characteristics of the patients with bulbar urethral stricture regarding length, type, diameter, and etiology have been shown in Table 1. Sixteen (20%) patients of bulbar urethral stricture developed recurrence after OIU and tri-inject. Recurrence occurred at 3 months in 9 (56.2%) patients and the remaining 7 (43.8%) developed within next 3 months. All patients with recurrence underwent another similar procedure, following which 4 developed recurrence whilst the remaining 12 patients were voiding well till the end of this study with at least three months of followup. Thus for bulbar urethral stricture, success rate of OIU and tri-inject was 80%, but short term success rate after two procedures reached 95%, with the success rate of the second procedure being 75%. On univariate analysis, history of previous OIU and length of stricture were not found to be of significance determining recurrence (*P* value of 0.13 and 0.059, resp.). All patients who had recurrence had diameter less than 6 Fr and none of the patients with wider residual lumen (i.e., more than 6 Fr) developed recurrence.

Seven patients had stricture localised to pendular urethra. Three patients had BXO, another three had history of catheterisation or instrumentation, and in one case no cause was found. None of these patients had history of any urethral surgery for stricture disease before presenting to us. In five patients, the length of the stricture was less than 2 cm and in 2 patients it was around 2-3 cm. In six patients the calibre of the residual lumen was more than or equal to 6 Fr and in one patient it was less than 6 Fr. All patients were kept on urethral catheter for 14 days after procedure. None of these patients developed a recurrence till the end of this study.

Sixteen patients with pan-anterior urethral stricture were treated by this modality. Ten patients (62.5%) had a history of instrumentation in the past. Four patients (25%) had BXO and two patients (12.5%) had history of UTI/STD. All patients were kept on per urethral catheter for 21 days after procedure. Four patients (25%) developed recurrence after the procedure within 3 months. All four underwent a repeat procedure after which again 2 (50%) developed recurrence within 3 months. Thus by the end of the study, the overall short term success after one or two procedures was 87.5%.

Combining the data of bulbar, pendular, and pan-anterior urethral stricture, the overall recurrence rate after the first OIU is 19.4% (20 out of 103 patients), that is, a success rate of 80.6%. Recurrence rates were, however, not found to be statistically significant between these three groups. Overall recurrence rate after second procedure was 5.8% (6 out of 103 patients), that is, a success rate of 94.2%.

All patients tolerated the therapy and none had local or systemic side effects of the injection.

## 4. Discussion

Mitomycin C is an antitumor antibiotic isolated from* Streptomyces caespitosus*. It has been found to inhibit fibroblast proliferation and prevent scar formation [10, 11]. Ayyildiz et al. have shown antifibrotic effect of MMC on experimentally induced urethral stricture in rats [12]. Mazdak et al. in 2007 reported study on 40 patients who were treated with urethrotomy with and without mitomycin C. Recurrence was seen in 2 out of 20 patients (10%) in mitomycin C group and 10 out of 20 patients (50%) in the other group [7]. Vanni et al. reported study on 17 patients of bladder neck contracture who were treated with bladder neck incision and intralesional MMC [13]. At a median followup of 12 months (range 4 to 26), 13 patients (72%) had a patent bladder neck after 1 procedure, as did 3 (17%) after 2 procedures and 1 after 4 procedures. All of the patients presenting with a prior indwelling urethral catheter or requiring a dilation schedule had a stable, patent bladder neck. Mazdak et al. in 2010 reported study on 25 patients treated by internal urethrotomy and intraurethral submucosal triamcinolone injection [14]. Recurrence was seen in 5 out of 24 patients (21.7%) and among 21 patients treated only by internal urethrotomy recurrence was seen in 11 patients (50%). Kumar et al. in 2012 studied 50 patients of stricture <3 cm treated with holmium laser with intralesional triamcinolone (80 mg) under spinal anesthesia. The overall recurrence rate was 24%. The success rate in patients with strictures less than 1 cm in length was 95.8%, whereas that in patients with strictures of 1 to 3 cm in length was 57.7% (*P* = 0.002) [6]. Tabassi et al. studied 70 patients of urethral stricture who were treated with internal urethrotomy and intraurethral triamcinolone injection. Recurrence was noted in 12 patients out of 34 and in 15 patients out of 36 in control group. No statistically significant difference was noted in recurrence rate (*P* = 0.584) but time to recurrence decreased significantly in experimental group (8.08 ± 5.55 versus 3.6 ± 1.59 months) (*P* < 0.05) [15].

Chung et al. studied 120 patients who underwent OIU for urethral stricture. Recruited patients were randomly divided into two groups: groups A and B. Patients in group A (60 patients) received HA/CMC (hyaluronidase and carboxymethylcellulose) instillation and patients in group B (60 patients) received lubricant instillation after internal urethrotomy [8]. Among 120 initial participants, 53 patients in group A and 48 patients in group B had completed the experiment. The recurrence of urethral stricture was observed in 5 cases (9.4%) in group A and in 11 (22.9%) in group B (*P* = 0.029). The mechanism is not clearly known in treatment of urethral stricture but hyaluronidase ameliorates pulmonary fibrosis and is also used in treatment of hypertrophic scar and keloid. The mechanism is supposed to be recruitment of autologous MSC-like cells to the lungs and decrease of TGF-b production and collagen deposition [16]. In treatment of hypertrophic scar and keloid, intralesional injection decreases fibroblast proliferation, collagen, and glycosaminoglycan synthesis and suppresses proinflammatory mediators in wound healing process [9].

In older series, the primary success rate of internal urethrotomy alone is around 60–70% [2]. In our study, use of all these three agents had an additive effect with success rate of 80.6% after the first OIU and 90.4% after the second procedure that was required in only 6 patients out of 103 patients. Thus the use of OIU in combination with tri-inject has encouraging success rate in the treatment of short segment anterior urethral stricture.

## 5. Conclusion

Combining the data of bulbar, pan-anterior urethral, and pendular stricture, the overall recurrence rate after the first OIU is 19.4% (20 out of 103 patients), that is, a success rate of 80.6%. Recurrence rates were, though, not found to be statistically significant between these three groups. Overall recurrence rate after second procedure was 5.8% (6 out of 103 patients), that is, a success rate of 94.2%. Thus optical internal urethrotomy with intralesional injection of Vatsala-Santosh PGI tri-inject (triamcinolone, mitomycin C, and hyaluronidase) is a safe and effective minimally invasive therapeutic modality for short segment anterior urethral strictures.

The limitation of the study is its noncomparative nature. However as compared to results of OIU in old studies [2], addition of intralesional tri-inject has encouraging results. The results need to be further verified in a randomised control trial involving a larger cohort.

## Figures and Tables

**Table 1:** Distribution of recurrence with respect to various characteristics of bulbar urethral stricture.

Characteristic	Subtype	Number of patients (percentage)	Recurrence (percentage)
Length	<2 cm	55 (68.750%)	8 (14.5%)
	2–4 cm	17 (21.25%)	4 (23.52%)
	>4 cm	8 (10%)	4 (50%)

Type	Primary	54 (67.5%)	8 (14.8%)
	Secondary	26 (32.5%)	8 (30.76%)

Diameter	<6 Fr	18 (22.5%)	16 (88.88%)
	≥6 Fr	62 (77.5%)	0 (0%)

Etiology	Known	39 (48.75%)	9 (23.07%)
	Idiopathic	41 (51.25%)	11 (26.82%)
